# Storage Mite Precision Allergy Molecular Diagnosis in the Moderate-to-Severe T2-High Asthma Phenotype

**DOI:** 10.3390/ijms23084297

**Published:** 2022-04-13

**Authors:** Ruperto González-Pérez, Paloma Poza-Guedes, Fernando Pineda, Miriam Castillo, Inmaculada Sánchez-Machín

**Affiliations:** 1 Allergy Department, Hospital Universitario de Canarias, 38320 Tenerife, Spain; pozagdes@hotmail.com (P.P.-G.); zerupean67@gmail.com (I.S.-M.); 2 Severe Asthma Unit, Hospital Universitario de Canarias, 38320 Tenerife, Spain; 3 Diater Laboratories, 28919 Madrid, Spain; f.pineda@diater.com (F.P.); m.castillo@diater.com (M.C.)

**Keywords:** airborne allergens, allergen exposure, asthma, storage mites, T2 inflammation

## Abstract

Storage mites (SM) may induce allergic respiratory symptoms in sensitized individuals, in both rural and urban settings. The relationship among specific IgE reactions to determined groups of SM allergens in the coincident asthma pheno-endotypes has not yet been investigated. We aimed to study a Precision Allergy Molecular Diagnosis (PAMD@) model to depict the SM molecular profile in individuals presenting with Type-2 inflammation, in two different (moderate and severe) asthma phenotypes. A customized PAMD@ panel, including SM allergens and their concurrent protein allergenic characterization was investigated. Mite group 2 allergens were most frequently recognized, including Lep d 2 (83.45%), followed by Gly d 2 (69.17%) and Tyr p 2 (47,37%), in 133/164 asthmatic subjects. Blo t 5 and Blo t 21 exhibited significant higher titres in both asthma groups. Although relevant mite group 2 allergens cross-reactivity is suggested, individualized sensitization patterns were relevantly identified. The present PAMD@ panel confirmed the dominance of mite group 2 allergens in moderate-to-severe T2 asthmatics. A broadly heterogeneous molecular repertoire of SM allergens was found in all subjects, regardless of their asthma severity. *Blomia tropicalis* deserves special attention in certain territories, as diagnostic and/or therapeutic approaches merely based on *Pyroglyphidae* mites may be insufficient.

## 1. Introduction

Asthma is a chronic and heterogeneous inflammatory lung disease, in terms of severity, natural history, and treatment responsiveness, reflecting the underlying pathogenic mechanisms and a highly prevalent cause of reduced quality of life in individuals of all ages, turning into a major public health and global economic burden due to both direct and indirect costs [1,2,3]. Asthma management can be individualized, grouping patients according to observable combinations of clinical, biological, and physiological characteristics into distinct pheno-endotypes [4,5], opening up the opportunity for new pathway precision diagnosis and targeted treatments [6,7]. Despite a broad number of distinct cell types that may play a unique role in the immunopathobiology of bronchial asthma, inflammatory cellular profiles of asthma principally fall into a dichotomy of type 2 response (resulting in eosinophilic inflammation) and non-type 2 response (thus, reinforcing non-eosinophilic, pauci-granulocytic inflammation) [8,9]. In addition, the upregulation of type 2 inflammation is characterized by an stimulus at the level of the airway epithelium that results in the production of alarmins -IL-25, IL-33, and thymic stromal lymphopoietin (TSLP) that stimulate the release of IL-4, IL-5, and IL-13, to activate and potentiate the innate and humoral arms of the immune system that lead to the pathogenic airway remodeling in asthma [10,11,12]. In addition, the interaction between the airway epithelium and the inhaled environment is crucial to understanding the pathogenesis of asthma, ever since the early demonstration that type-I hypersensitivity to dust-mite-derived allergens and adjuvants were the origin of the allergenicity of house dust [13]. The distribution of mite species in a geographical area is markedly affected by the local climate conditions, and diverse types of mites dominate in different regions of the world [14]. In this regard, numerous mite species have been described capable of sensitizing and inducing clinical manifestations in humans, in sensitized and genetically predisposed subjects [15,16,17]. Therefore, mites are generally grouped into house dust mites (HDM) belonging to the *Pyroglyphidae* family and storage mites (SM) (also known as grain mites, flour mites, and/or forage mites), including *Acaridae* and *Glycyphagydae* families, related to rural occupational exposure and humid urban dwellings [18,19,20]. The term “domestic mites” refers to mite species present in the indoor environment, causing the development of specific immunoglobulin E (sIgE) antibody responses, encompassing both HDM and SM categories. This is the case for *Blomia tropicalis* (*B. tropicalis*), formerly known as an SM, which is nowadays considered a domestic mite, extensively present in homes from tropical and subtropical countries [21,22]. Although several SM allergens have been recently purified, cloned and sequenced, the variations in the biological activity among allergen extracts and manufacturers may restrict the full understanding of the in vivo relevance of the sensitization to these molecules [23,24].

Another issue of concern in the published literature is that SPT reactivity to SM can either represent “true positivity” or “false positivity” due to their taxonomic proximity with HDM [25,26]. In fact, mites of the same family share several proteins with great homology, showing a high cross-reactivity among the *Glycyphagydae* family and a moderate reactivity between *B. tropicalis* and *Tyrophagus putrescentiae* (*T. putrescentiae*) [27]. To overcome this situation, the current availability of DNA sequences of allergens has allowed the preparation of purified, recombinant allergens and hypoallergenic allergen derivatives, that can be used for Precision Allergy Molecular Diagnosis (PAMD@) to identify the individual molecules, to elucidate between actual poly-sensitization to numerous allergens and cross-reactivity [28]. Despite HDM exposure and sensitization having been previously documented as strong predictors for respiratory disease, there is still limited evidence concerning SM-sIgE responses taking part in the pathogenesis of the T2 inflammation asthma phenotype [29,30,31]. Moreover, data are scarce for subjects simultaneously sensitized to HDM and SM. Here, we investigated a personalized PAMD@ model approach to depict the molecular profiling of different SM families from selected non-occupational moderate and severe asthmatics with the distinct T2-phenotype, subjected to the influence of perennial subtropical weather conditions.

## 2. Results

### 2.1. Demographic Characteristics of Patients

**Figure 1:**
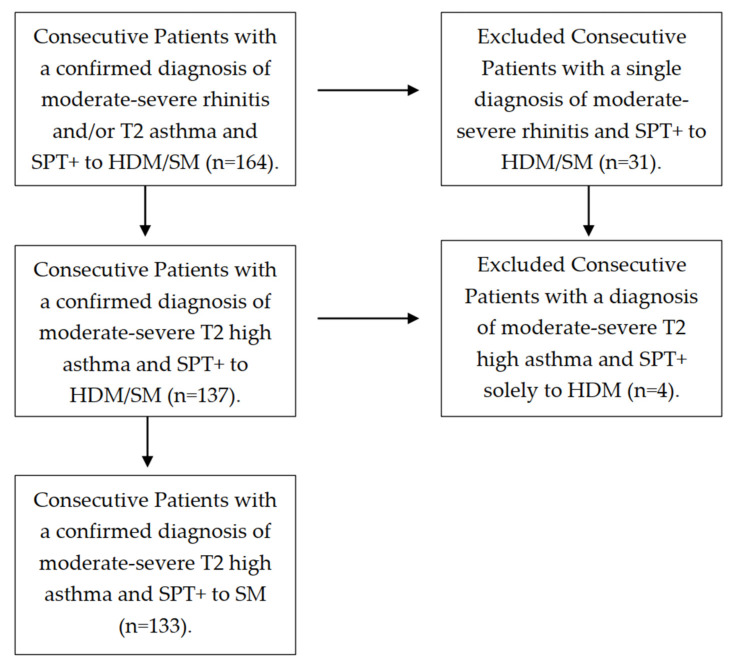
Flow diagram of study selection.

A total of 164 patients were screened, with 133 of them—79 females, 54 males, median age 26.0 years old—finally confirming their eligibility for the study (Figure 1). All subjects who fulfilled the GINA criteria [32] for moderate-to-severe non-occupational T2 persistent asthma showed a positive SPT to any SM. Most of the subjects (109 out of 133 patients, 82.56%) had their asthma onset during childhood or adolescence. All subjects were on regular daily treatment—comprising both mite avoidance measures and medical therapy—according to their asthma stage and severity. Five out sixty-two (8.06%) patients from the severe asthma cohort were on oral steroids upon inclusion in the study. Regarding atopic comorbidities, 121 patients (90.11%) suffered from allergic rhinitis, 32 subjects (24.06%) had atopic dermatitis and 23 (17.29%) had food allergy (seafood, nuts, egg and/or milk) associated. The majority of patients (86.66%) reported a known family history of atopy.

### 2.2. Total IgE and Blood Eosinophils

A quantitative analysis of serum total IgE was performed in order to evaluate the basal atopic status in the study population. The total IgE showed a median value of 642 IU/mL. The severe asthmatic patients showed a median total IgE value (661 IU/mL) slightly higher than the moderate asthma group (635.5 IU/mL). Blood eosinophils showed a median value of 400 eosinophils/μL, again with a higher significant (*p* < 0.05) median value (420 eosinophils/μL) in the severe asthma group, with respect to those with the moderate asthma (395 eosinophils/μL) phenotype (Table 1).

### 2.3. Prevalence, Sige Reactivity and Individual Molecular Profile in Serum from SM Asthmatic Subjects

Sensitization to mite extracts by SPT and the prevalence of 133 patients who met the inclusion criteria are summarized in Table 2.

**Figure 2:**
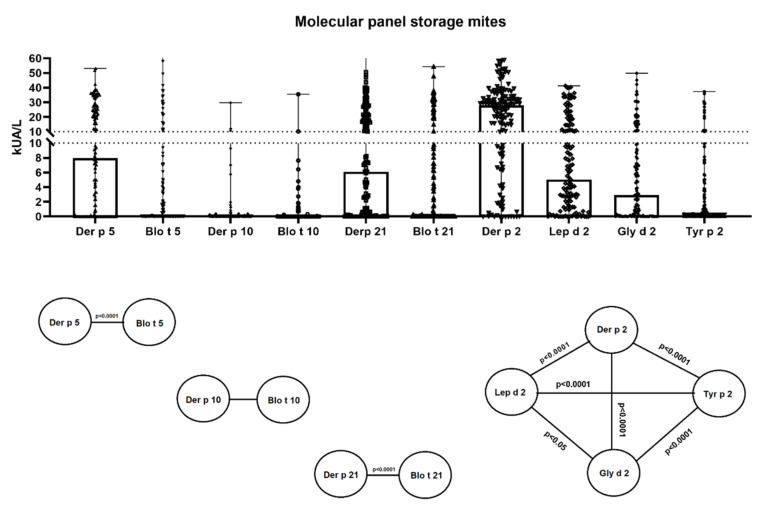
Specific IgE serodominance in a comprehensive panel of molecular allergens to *Dermatophagoides pteronyssinus, Blomia tropicalis, Lepidoglyphus destructor, Glyciphagus domesticus* and *Tyrophagus putrescentiae* in 133 asthmatic subjects. Statistical differences (*p* < 0.001) among mite allergens from groups 2, 5 and 21 are shown.

One hundred and twenty-one patients (90.97%) were independently sIgE positive (≥0.35 kU_A_/L), for either the whole extract of *B. tropicalis*, *L. destructor, A. siro*, *G. domesticus*, and/or *T. putrescentiae* and/or one of the following individual molecules: Blo t 5, Blo t 10, Blo t 21, Lep d 2, Gly d 2 and/or Tyr p 2. Sensitization to the complete *B. tropicalis* and *L. destructor* extract was confirmed in 76 (57.14%) and 62 (46.61%) patients, respectively, followed by a serum sIgE response to the *A. siro* extract in 57 (42.85%) individuals, *T. putrescentiae* in 39 cases (29.32%) and *G. domesticus* in 30 subjects (22.55%). Only 2 subjects out of 133 (1.5%) with a positive sIgE response to SM were not sensitized to sIgE-*D. pteronyssinus* (whole extract). Considering molecular allergens exclusively, group 2 was most frequently identified, with sIgE positive for Lep d 2 in 111 out of 133 subjects (83.45%), followed by Gly d 2 (69.17%) and Tyr p 2 (47.37%). The mean value of sIgE (kU_A_/L) against Lep d 2 (10.08), was also significantly (*p* < 0.05) higher than Gly d 2 (7.82) and Tyr p 2 (3.51). Concerning *B. tropicalis* major allergens, Blo t 5 (mean sIgE 6.09 kU_A_/L) was found in 58 asthmatics (43.6%), followed by Blo t 21 (mean sIgE 7.09 kU_A_/L) in 51 patients (38.34%), while Blo t 10 (mean sIgE 0.63 kU_A_/L) was limited to 19 subjects (14.28%). Statistical differences (*p* < 0.001) were found among HDM and SM allergens from groups 2, 5 and 21 (Figure 2).

### 2.4. Relation of Sige-Sensitization Profile and Severity of Asthma

**Figure 3:**
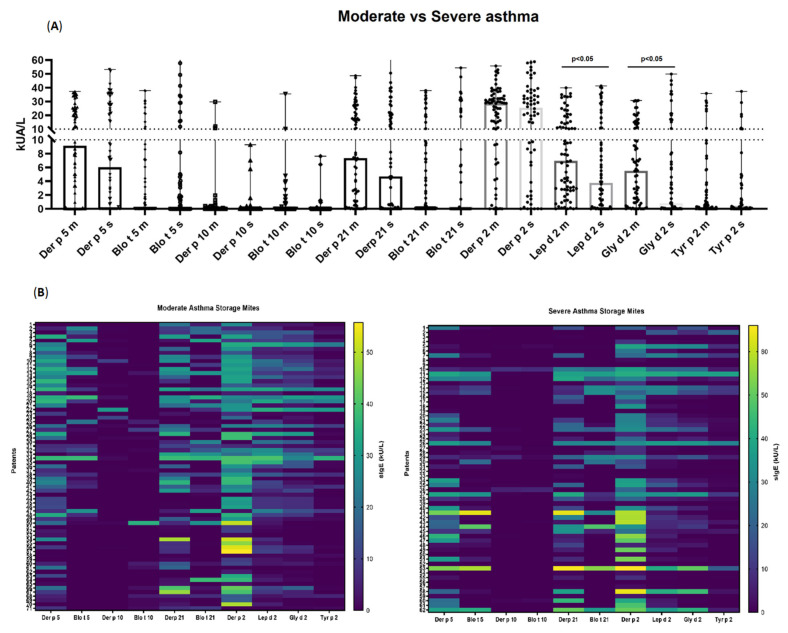
Sensitization profile to specific IgE (sIgE) (**A**) and heatmap (**B**) to a comprehensive panel of molecular allergens of *Dermatophagoides pteronyssinus, Blomia tropicalis, Lepidoglyphus destructor, Glyciphagus domesticus* and *Tyrophagus putrescentiae* in 133 moderate (m) to severe (s) asthmatic patients. Broadly different patterns of sIgE were identified in both groups of asthmatics.

Specific IgE (kU_A_/L) to the whole extract of *B. tropicalis* was higher in the moderate asthma group (22.21 ± 7.35) compared to the severe phenotype (16.87 ± 9.22). In contrast, severe asthmatics showed slightly higher mean sIgE to complete *G. domesticus*, *A. siro* and *T. putrescentiae* extracts, compared to moderate asthmatics. With respect to individual molecules, non-significant (*p* = 0.48), higher sIgE titres were found for Blo t 5 in the severe asthma group (7.42 ± 5.67), compared to the milder (4.77 ± 3.86) asthma patients. Higher quantitative differences (*p* < 0.05) were only found for Lep d 2 and Gly d 2 in the moderate asthmatics, with no further variations in the rest of the investigated molecules (i.e., Tyr p 2, Blo t 10 and Blo t 21), as displayed in Figure 3.

### 2.5. Molecular Sensitization Profiles and Aggregation into Mite Allergen Homologous Proteins

Lep d 2 exhibited the most prevalent individual SM allergen in 17 out of 21 (77.77%) different depicted SM profiles, followed by Gly d 2 (59.25%), Blo t 5 (48.14%), Bl t 21 (44.44%), Tyr p 2 (37.03) and Blo t 10 (37.03%). Nineteen patients (14.28%) showed a monomolecular sIgE response to either Lep d 2 (11.27%), Gly d 2 (1.5%), Blo t 5 (0.75%) or Blo t 10 (0.75%). Meanwhile, no subjects were found to be solely sensitized to Blo t 21. In contrast, despite 14 subjects (10.52%) having a positive sIgE response to the crude extract of SM, no detection to any of the 6 individual available SM molecular allergens was found. Considering the principle of homologous groups among *D. pteronyssinus* and SM, the repertoire of recognized molecules (sIgE against groups 2, 5, 10 and 21) was markedly complex, including 41 distinct profiles in 133 subjects (Table 3), with 8 specific molecules (Der p 2, Lep d 2, Gly d 2, Tyr p 2, Der p 5, Blo t 5, Der p 21, and Blo t 21), most frequently (15.03%) identified in both groups of asthmatics. Further, 6 out 133 patients (4.51%) were sensitized to either Lep d 2, Gly d 2 and/or Tyr p 2, but not to Der p 2. In addition, nine patients (6.76%) showed a positive sIgE response to Blo t 10 but not to Der p 10, while eight subjects (6.01%) were selectively sensitized to Blo t 5 and not to Der p 5, and five individuals (3.75%) were sensitized to Blo t 21 but not to Der p 21.

### 2.6. SDS PAGE and IgE Western Blot

**Figure 4:**
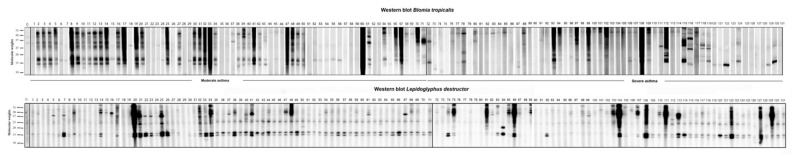
IgE Western blot of selected patients for *Blomia tropicalis* and *Lepidoglyphus destructor* allergenic extract. A marked IgE-binding intensity was found around 13, 14, 17, 30, 35, 50 and 55 kDa for *Blomia tropicalis* in the majority of subjects, and around 14–16 kDa mainly for patients with mild–moderate asthma and a more heterogenous repertoire including 14–16, 25, 40 and 50 kDa for *Lepidoglyphus destructor* in severe asthmatics.

Western blot of selected patients with a sensitization to *B. tropicalis* and *L. destructor* showed different patterns of sensitization (Figure 4). A marked IgE-binding intensity was found around 13, 14, 17, 30, 35, 50 and 55 kDa for *B. tropicalis* in the majority of subjects, and around 14–16 kDa for patients with moderate asthma with a more complex repertoire, including 14–16, 25, 40 and 50 kDa for *L. destructor* in those with the severe asthma presentation.

## 3. Discussion

Unique attributes of mites have allowed them to colonize the indoor environment, producing an unparalleled diversity of allergens and adjuvants, perfectly complemented to elicit both innate and adaptive immune reactions [33]. In fact, despite SM having been traditionally related to occupational disease, attention has been focused on their clinical relevance in the adult general population and children in non-occupational environments, from temperate regions of the world [34,35,36]. Conforming to our previous observations, describing the clinical role of SM in different atopic conditions, most of the screened SM patients (98.5%) were simultaneously sensitized to HDM [37,38]. Studies of cross-reactivity among non-pyroglyphid mites have been carried out, with conflicting results, showing a limited cross-reactivity among Gly d 2, Lep d 2, and Tyr p 2 [39]. The 14-kDa mite group 2 allergen—belonging to the NPC2 (NPC intracellular cholesterol transporter 2, Niemann-Pick proteins type C2) family—was identified in more than 83% of the current cohort, with Lep d 2 as the most frequently SM-depicted allergen, closely followed by Gly d 2 and Tyr p 2, to a lesser extent [40]. In line with previous reports [41], indicating an important IgE cross-reactivity among Lep d 2, Gly d 2 and Tyr p 2, and a partial cross-reactivity between *T. putrescentiae* and *G. domesticus*/*L. destructor*, we found that more than 50% of the individuals were all concomitantly sensitized to these three molecules, regardless of the asthma severity.

It is noteworthy that, despite Gly d 2 sharing a high (79%) sequence homology with Lep d 2 [42], only 31.98% of individuals presented a selective response to Lep d 2 and Gly d 2, followed by Gly d 2 and Tyr p 2 (3.36%) and Lep d 2 and Tyr p 2 (1.68%). Further, in accordance with reports from Southern Spain, nearly all patients (>98%) with a positive response to Gly d 2 were also sensitized to Lep d 2, whereas this mite has been detected in only 2.6% of the patients’ house dust samples [43,44]. Interestingly, both Lep d 2 and Gly d 2 may be speculated as potential biomarkers in milder forms of asthma presentations, as significant differences were only found in the moderate asthmatics against the severe asthma group. In respect of group 2 allergen interactions, above 90% of the screened subjects showed a sIgE response to Der p 2, and more than half of them were concurrently sensitized to Lep d 2, Gly d 2 and Tyr p 2. In agreement with the low (45%) sequence homology described for group 2 proteins between *D. pteronyssinus* and SM [45,46], only 16.52% of subjects were sensitized to Der p 2 and Lep d 2, with even lower ratios between Der p 2 and Gly d 2 (1.65%) and Der p 2 and Tyr p 2 (0.82%).

Molecular sensitization to *B. tropicalis* was broadly present in the selected cohort (63.02%), supporting a pivotal role for Blo t 5, not only in respiratory allergies, but also in the severity of the underlying disease [47,48]. These findings may be of interest, as murine models of CD4 T-cell epitopes for Blo t 5 could be therapeutically employed to suppress the inflammatory response in the allergic airway, regardless of the inferred gravity of symptoms [49]. 

Considering the homology between group 5 and 21 allergens, above 71% subjects showed sIgE to Der p 5 or Der p 21. Despite low cross-reactivity having been only addressed between group 5 allergens [50], similar ratios were found for patients concomitantly sensitized to Der p 5 and Blo t 5 (50%), and/or Der p 21 and Blo t 21 (46.31%).

Group 10 tropomyosins are noted for their conserved amino acid sequences, being almost identical to other arthropod tropomyosins and, thus, being involved in cross-reactivity mechanisms [51]. In the present study, not only was Blo t 10 more frequently identified than Der p 10, but also selective Blo t 10 responses were detected. Notably, although 10 asthmatic patients were associated with a confirmed—double-blind placebo-controlled challenge (DBPCC) according to their previous medical records—seafood allergy diagnosis, Blo t 10 and Der p 10 were only identified in three and two subjects, respectively.

Despite different patterns of sensitization to *B. tropicalis* having been outlined, the additional quantification of potentially relevant allergens from *B. tropicalis* is not commercially available at present. This is the case for Blo t 4, described as a local serodominant allergen, showing an unusually higher frequency than Blo t 5, in areas from China and Spain [52,53]. Further, recent progress in the immuno-characterization of *B. tropicalis*, has elegantly evidenced that Blo t 2 is a clinically relevant allergen, with unique IgE epitopes, compared to the major group 2 allergens from *Dermatophagoides* spp. [54].

Although the concept of homologous groups could serve as a dynamic tool in the regulation of allergen products, our findings do not support a reduction in the study panel of mite allergens, as mites other than *Pyroglyphidae* are frequently present in significant amounts in tropical and subtropical dust samples, from both rural and urban environments [55,56]. The current study showed several restrictions, as 14 subjects (10.52%) with a positive sIgE response to the raw extract of SM (with 3 and 2 out of those 14 patients showing a positive SPT to *G. domesticus* and *A. siro*, respectively) could not be identified through the proposed molecular panel, and also, a limited number of patients was studied, making these findings challenging to match with other SM-sensitive asthma cohorts, exposed to different mite populations.

## 4. Materials and Methods

### 4.1. Subjects

We consecutively recruited patients with a clinical diagnosis of moderate-to-severe persistent rhinitis and/or non-occupational asthma with the mixed T2 endotype (i.e., eosinophilic, high total IgE and sIgE to airborne allergens) according to the 2020 GINA Guidelines [32] from the Severe Asthma Unit and the Outpatient Allergy Clinic at Hospital Universitario de Canarias (Tenerife, Spain), serving an area of 368,000 inhabitants under subtropical climate conditions, and mites as the most frequently found respiratory allergen [57]. Asthma severity and staging were also clinically evaluated according to the aforementioned specific Guidelines [32].

Included subjects needed to achieve the subsequent clinical standards: persistent non-occupational respiratory symptoms with recurrent exacerbations, improvement in symptoms at altitudes (>1500 m) and increase in symptoms with household dust and indoor activities. The following clinical data were collected from the patients’ medical records: forced expiratory volume in the first second (FEV1), rhinitis and conjunctivitis diagnosis, treatments, a validated Asthma Control Test (ACT), and skin prick test (SPT) results. Only subjects with an immediate positive SPT to any SM extract were included. Blood samples were obtained from all participating individuals, identified with a code label, stored at −40 °C and thawed immediately prior to the in vitro assay. Patients under treatment with allergen immunotherapy or monoclonal antibodies (biologics) were excluded. Pregnant and breast-feeding women were also excluded. The investigation was approved by the local Ethical Committee of our institution and informed consent was signed by all subjects and parents/guardians for those participants <18 y.o.

### 4.2. Skin Prick Test

Percutaneous testing was carried out according to European standards [58], enclosing a diagnostic panel (Diater, Madrid, Spain) with standardized (*Dermatophagoides pteronyssinus (D. pteronyssinus)*, *B. tropicalis*, *Lepidoglyphus destructor* (*L. destructor*), and *T. putrescentiae*) and non-standarized (*Glycyphagus domesticus* (*G. domesticus*) and *Acarus siro* (*A. siro*)) mite extracts. Histamine (10 mg/mL) and saline were used as positive and negative controls as usual. Antihistamines were withdrawn a week before the SPT, and wheal diameters were immediately measured after 20 min with those diameters greater than 3 mm considered as positive.

### 4.3. Mite Allergenic Extracts

Proteins from mite bodies of *D. pteronyssinus*, *B. tropicalis*, and *L. destructor* were extracted in phosphate-buffered saline buffer (PBS), 0.01 M, pH 7.4, for 2 h at 5 ± 3 °C. Both protein solutions were clarified by filtration and centrifugation (1 h at 16,000× *g*). Afterwards, the isolated supernatants were ultrafiltered against highly purified water (Ph. Eur. specification), sterile filtered, frozen and lyophilized.

### 4.4. SDS PAGE and IgE Western Blot

Proteins from *B. tropicalis*, and *L. destructor* extracts were analyzed by sodium dodecylsulfate polyacrylamide gel electrophoresis (SDS-PAGE), according to Laemmli [59] in 15% polyacrylamide gels under reducing conditions. Proteins were visualized by Coomassie Brilliant Blue R-250 staining and transferred to polyvinylidene difluoride (PVDF, Trans-blot turbo TM. BIORAD, Hercules, CA, USA). The binding of IgE antibody to allergens was analyzed by Western blot using individual patients’ sera and anti-human IgE peroxidase conjugate (Southern Biotech, Birmingham, AL, USA). Chemiluminescence detection reagents (Western lightning^®^ Plus-ECL. Perkin Elmer. Waltham, MA, USA) were added following the manufacturer’s instructions. IgE-binding bands were identified using the BioRad Diversity database program.

### 4.5. Blood Eosinophils and Serological Analysis

Peripheral blood eosinophils were determined and expressed as eosinophils/μL. Total IgE levels, sIgE to *B. tropicalis, L. destructor, A. siro*, *G. domesticus*, *T. putrescentiae* and *D. pteronyssinus* (whole extract), and sIgE to Der p 2, Der p 5, Der p 10, Der p 21, Blo t 5, Blo t 10, Blo t 21, Lep d 2, Gly d 2, Tyr p 2 were measured (ALEX MacroArray Diagnostics, Vienna, Austria) according to the manufacturer´s instructions. In brief, ALEX is a multiplex array containing 282 reagents (157 whole allergens and 125 molecular components). The different allergens and components are coupled onto polystyrene nano-beads, and then the allergen beads are deposited on a nitrocellulose membrane, as formerly published [60]. Total IgE levels were expressed in international units per unit volume (IU/mL), sIgE levels were expressed in kU_A_/L. Values ≥ 0.35 kU_A_/L were considered positive.

### 4.6. Statistical Analysis

Demographic features were summarized by medians and standard deviations for continuous variables and percentages for categorical variables. To compare differences analysis of variance, Kruskal–Wallis, Mann–Whitney U and Chi-square tests are required for parametric continuous, nonparametric continuous, and categorical variables respectively. A P value of less than 0.05 was considered statistically significant. All statistical data were analyzed using GraphPad Prism version 8.0.0 for Windows, GraphPad Software, La Jolla, CA, USA.

## 5. Conclusions

The present study is, to our knowledge, the first to investigate a real-life molecular response to a comprehensive panel of SM allergens in our territory, displaying a widely heterogeneous repertoire in more than 89% of the T2 asthma subjects. Although the analysis of PAMD@ revealed the dominance of mite group 2 allergens, and part of the detected IgE signals suggest determined cross-reactivity, the depiction of individual sensitization patterns should be carefully evaluated for precise diagnosis and targeted AIT. Similarly, SM distinctly demands specific attention in certain regions, as current diagnostic and therapeutic tools based on *Dermatophagoides* allergens may lead to under-diagnosis or inaccurate treatment. In addition, the regular follow-up of SM sensitization could potentially be a non-invasive marker of epithelial barrier dysfunction in asthma, and to monitor other affected tissues [61]. The proposed PAMD@ panel approach ought to be included to provide a more precise diagnosis, particularly in populations with concomitant HDM and SM exposure, thus, expanding the intricacy of the molecular picture in this asthma phenotype. Furthermore, our results confirm that molecular diagnosis is a sensitive and highly specific tool to determine SM allergen exposure in certain territories. 

## Figures and Tables

**Table 1:** Descriptive statistics regarding basal comorbid conditions and associated asthma features in the studied population (n = 133).

	Moderate Asthma	Severe Asthma
n = 133	71 (53.38%)	62 (46.62%)
Age (y.o.)	29.59 ± 13.82	33.85 ± 16.05
<20 y.o. (n = 41)	19 ± 7.3 (26.76%)	16 ± 5.21 (25.8%)
>20 y.o. (n = 92)	52 (73.24%)	46 (74.2%)
Sex (F/M)	56.33%/43.67%	61.3%/38.7%
BMI	26.54 ± 5.9	28.59 ± 5.4
Allergic Rhinitis	64 (90.14%)	55 (88.7%)
Atopic Dermatitis *	17 (23.94%)	4 (6.45%)
Nasal Polyposis *	2 (2.81%)	8 (12.9%)
Food Allergy	9 (12.67%)	3 (4.83%)
NSAID sensitivity	2 (2.81%)	2
ACT	18 (16–25)	14 (9–17)
FVC	3728 (90.1 ± 13.56%)	3212 (79.0 ± 15.03%)
FVC < 20 y.o.	90.87 ± 15.67%	81.24 ± 17.22%
FEV1 *	3027 (91.32 ± 15.07%)	2475 (76.12 ± 17.41%)
FEV1 < 20 y.o.	92.33 ± 18.81%	81.92 ± 16.33%
SPT+HDM/SM	71 (100%)	62 (100%)
Total IgE (IU/mL)	635 ± 733	661 ± 702
Eosinophils/μL peripheral blood *	395 ± 225	420 ± 259
Asthma Onset at Childhood	53 (74.6%)	48 (77.41%)
Family History of Atopy	59 (83.09%)	51 (82.25%)

SPT: Skin Prick Test. SM: Storage mites. BMI: Body Mass Index. ACT: Asthma Control Test. FVC: Forced Ventilatory Capacity. FEV1: forced expiratory volume in the first second. Median values and standard error of the median (SEM) are shown. * Indicates statistical significance (*p* < 0.05).

**Table 2:** Prevalence of sensitization to mites by Skin Prick Test.

Skin Prick Test	N (%)
*Dermatophagoides pteronyssinus*	131 (98.5)
*Blomia tropicalis*	91 (68.42)
*Lepidoglyphus destructor*	68 (51.12)
*Acarus siro*	61 (45.86)
*Tyrophagus putrescentiae*	46 (34.58)
*Glycyphagus domesticus*	36 (27.06)

**Table 3:** Specific IgE profiles aggregated into mite homologous group allergens 2, 5, 10 and 21 between *D. pteronyssinus* and storage mites in 133 subjects tested with microarray. Profiles are ordered by the increasing number of recognized molecules.

n = 133	%	Number of Molecules	Der p 2	Lep d 2	Gly d 2	Tyr p 2	Der p 5	Blo t 5	Der p 10	Blo t 10	Der p 21	Blo t 21
4	3.00	0										
4	3.00	1	*									
1	0.75	1					*					
1	0.75	1									*	
2	1.50	2	*	*								
1	0.75	2	*				*					
2	1.50	2	*								*	
1	0.75	2	*		*							
3	0.75	3	*	*			*					
2	1.50	3	*				*				*	
1	0.75	3		*	*	*						
1	0.75	3					*			*	*	
1	0.75	3		*	*						*	
2	1.50	3	*	*							*	
1	0.75	3	*	*					*			
1	0.75	3	*		*				*			
3	2.25	4	*	*	*		*					
1	0.75	4	*	*	*	*						
1	0.75	4	*				*	*			*	
1	0.75	4	*	*			*			*		
6	4.51	4	*	*			*				*	
2	1.50	4	*			*		*				*
3	2.25	4	*	*	*						*	
2	1.50	4	*	*	*		*					
1	0.75	4	*	*		*	*					
1	0.75	4	*	*						*	*	
3	2.25	5	*	*	*		*				*	
3	2.25	5	*	*	*						*	*
2	1.50	5	*	*	*	*	*					
1	0.75	5	*	*	*	*					*	
1	0.75	5	*	*			*				*	*
1	0.75	5	*	*				*			*	*
1	0.75	5	*	*			*	*			*	
1	0.75	5		*	*			*		*		*
9	6.76	6	*	*	*	*	*				*	
7	5.26	6	*	*	*		*	*			*	
2	1.50	6	*	*	*		*		*	*		
1	0.75	6	*	*	*	*	*			*		
1	0.75	6	*	*	*		*				*	*
1	0.75	6		*			*	*		*	*	*
1	0.75	6	*	*	*		*				*	*
1	0.75	6		*	*		*			*	*	*
1	0.75	6	*	*			*	*			*	*
1	0.75	6	*	*	*	*			*			*
8	6.01	7	*	*	*	*	*	*			*	
5	3.75	7	*	*	*	*	*				*	*
1	0.75	7	*	*	*	*	*	*				*
1	0.75	7	*	*	*	*	*	*	*			
1	0.75	7	*	*	*	*	*		*		*	
1	0.75	7	*	*	*	*		*			*	*
1	0.75	7	*	*	*		*		*	*	*	
1	0.75	7	*	*	*		*	*			*	*
20	15.03	8	*	*	*	*	*	*			*	*
1	0.75	8	*	*			*	*	*	*	*	*
3	2.25	9	*	*	*	*	*	*	*		*	*
2	1.50	9	*	*	*	*	*	*		*	*	*
1	0.75	10	*	*	*	*	*	*	*	*	*	*

Asterisk (*) indicates specific IgE sensitization to a single mite molecular allergen.

## Data Availability

The data that support the findings of this study are available from Servicio Canario de Salud but restrictions apply to the availability of these data, which were used under license for the current study, and so are not publicly available. Data are, however, available from the authors upon reasonable request and with the permission of Servicio Canario de Salud.
